# Supplementary material for: Melatonin Rescues Dimethoate Exposure-Induced Meiotic and Developmental Defects of Porcine Oocytes
Source: Animals (Basel). 2022 Mar 25;12(7):832. doi: 10.3390/ani12070832 (PMC8997005; doi:10.3390/ani12070832)
Supplement: Supplementary file 1 [file animals-12-00832-s001.zip › animals-1617318-supplementary.pdf]

Table S1. Information on primary and secondary antibodies used in this study

Antibody	Species	Vendor	Cat.no.and dilution
γH2AX	Mouse	Abcam	ab26350（1:200）
α-Tubulin	Mouse	Sigma	F2168（1:200）
LC3B	Rabbit	Cell Signaling	2775S (1:200)
Alexa Fluor 594 anti-mouse IgG	Goat	Invitrogen	A11005 (1:200)
Alexa Fluor 488 anti-rabbit IgG	Goat	Invitrogen	A11008 (1:200)
Alexa Fluor 594 anti-rabbit IgG	Goat	Invitrogen	A11012 (1:200)

Information on reagents used in this study

Components	Vendor	Cat.no.
Phalloidin, Fluorescein Isothiocyanate Labeled	Sigma	P5282
MitoTracker™ Red CMXRos	Invitrogen	M7512
Lectin from Arachis hypogaea (peanut)	Sigma	L7381
Reactive Oxygen Species Assay Kit	Beyotime Biotechnology	S0033
